# Keystone Veterinary Medical Association

**Published:** 1900-01

**Authors:** E. M. Ranck

**Affiliations:** Secretary pro tem.


### SOCIETY PROCEEDINGS.

### KEYSTONE VETERINARY MEDICAL ASSOCIATION.

THE regular monthly meeting was held at 8 P.M., December 12, 1899, in the reception-room of the Odd Fellows' Temple. In the absence of the President, Dr. Thomas B. Rayner was called to the chair, and Dr. E. M. Ranck was appointed Secretary *pro tem.* in the absence of Secretary Rhoads. The following members of the profession were present: Drs. T. B. Rayner, Williams, Underhill, Marshall, Pearson, Hoskins, Schneider, Harger, Rhoads, Lintz, Drake, and Ranck. Dr. J. C. Morris, honorary member, was also present.

The programme consisted of the discussion of milk-inspection and reports of cases. The discussion on milk-inspection was opened by Dr. Ranck on the needs of a more thorough system of inspection. Dr. Hoskins followed with remarks favorable to the delivery of milk in sealed jars or bottles when so prepared at the source of production. Dr. Leonard Pearson addressed the Association at some length on ways and means to educate the public to the appreciation of a better milk-supply, suggesting that this may first be aided through the medical profession.

(*a*) By veterinarians writing papers including the necessities for better milk-supply, the paper published, and reprints sent to all physicians.

(*b*) Issuing a regular bi-weekly ' milk paper,'' to be distributed broadcast, especially among the medical profession.

(*c*) By certain forms of letters (personal) to the physicians.

(*d*) By personal demonstration of facts regarding the dangers through an inefficient milk-inspection as well as the proper manipulation of the product; also through that class of people who are in favor with the movement tending to better the health condition. Through the farmer by making his product worth more in the market.

Dr. J. Cheston Morris addressed the Association from the position of one who had dealt with the question from a productive stand-point for more than twenty years. He thought the public much better informed and more aroused to the importance of a wholesome milk-supply and to the dangers incidental thereto than they had ever been before, and the best way to get a better article is through the pockets of the producer; as his returns increase so can the quality of the milk; by contributing to the pages of local papers and the discussion of the subject a valuable plan to arouse interest and just rivalry among the producers. He drew attention to the subjoined interesting facts:

1. That the average milk-supply for each man, woman, and child in a number of Eastern cities is from two-fifths to one-half pint, and careful investigation shows that it should be at least one quart.

2. That two quarts of milk and one-half loaf of bread contain more nutriment than the ration of the French Army.

3. That one quart of milk contains more nutriment than one pound of meat.

Further remarks were made by Drs. C. J. Marshall, Williams, and others.

The discussion closed with the appointment of a committee to take the matter in charge for further consideration at the January meeting.

Reports of cases being in order, Dr. Leonard Pearson reported an outbreak of a disease, apparently specific in character, infectious and contagious, resembling influenza in a mild type, with one death, that exhibited on post-mortem small areas of pneumonia, but not sufficiently extended to cause death; the animal exhibited rise in temperature, increased pulse; but one exhibited a cough, marked depression, and a very light per cent. of the horses in the stable escaping. The cases under the charge of Dr. Williams were very light and slow to respond to a varied line of treatment. The use of distemper antitoxin was suggested in connection with general stimulating treatment. This was done with apparent good effect in a number of cases.

Dr. Hoskins reported a number of diabetes insipidus, or polyuria, in a number of horses, with a detailed statement of the chemical tests of the urine, the same showing as low a specific gravity as 1003 to 1007, the absence of albumin or sugar and alkaline in character. The use of sanmetto, combined with nux vomica, gave permanent and gratifying results, with no disposition to recurring attacks.

President Rayner appointed the following committee to consider and further the milk question: Drs. Pearson, Hoskins, Ranck, Marshall, and Rhoads, after which the meeting adjourned.

E. M. RANCK,
Secretary pro tem.

